# Effects of rapid maxillary expansion on auditory thresholds and middle ear functioning

**DOI:** 10.1590/2317-1782/e20230343en

**Published:** 2025-01-27

**Authors:** Cassiele Fontoura Moraes, Erissandra Gomes, Rebeca Cardona Santa Helena, Pricila Sleifer

**Affiliations:** 1 Universidade Federal do Rio Grande do Sul – UFRGS - Porto Alegre (RS), Brasil.; 2 Faculdade de Odontologia, Universidade Federal do Rio Grande do Sul – UFRGS - Porto Alegre (RS), Brasil.; 3 Universidade Federal do Rio Grande do Sul – UFRGS - Porto Alegre (RS), Brasil.; 4 Instituto de Psicologia, Serviço Social, Saúde e Comunicação Humana, Universidade Federal do Rio Grande do Sul – UFRGS - Porto Alegre (RS), Brasil.

**Keywords:** Hearing, Hearing Loss, Palatal Expansion Technique, Maxilla, Children, Adolescents

## Abstract

**Purpose:**

To ascertain whether Rapid Maxillary Expansion (RME) elicits effects on the functioning of the middle ear and air-bone gaps in children and adolescents.

**Methods:**

Single-arm clinical trial, with data collection at four time points: before initiating Rapid Maxillary Expansion (RME) (T0), upon completion of RME (T1), three months post-RME completion (T2), and six months post-RME procedure (T3). The audiological assessment, conducted at all four time points, comprised otoscopy, pure tone and speech audiometry, tympanometry, and acoustic reflex investigation.

**Results:**

Eighteen children and adolescents met the eligibility criteria. There was a reduction in air-bone gaps and an improvement in middle ear function throughout the follow-up period, between T0, T1, T2, and T3. Three months after the completion of RME, at T2, all patients exhibited type A tympanometric curves, and six months after RME, at T3, there was an absence of air-bone gaps and ipsilateral and contralateral acoustic reflexes present in the entire sample.

**Conclusion:**

In the studied sample, it was observed that Rapid Maxillary Expansion (RME) led to a gradual reduction in air-bone gaps, resulting in appropriate middle ear function in children and adolescents with transverse maxillary atresia.

## INTRODUCTION

Transverse maxillary atresia is a dentofacial anomaly characterized by a reduction in the transverse diameter of the maxillary arch^(1,2)^. The main etiological factors of this deficiency include mouth breathing, deleterious habits such as thumb sucking and/or pacifier use, adaptive/atypical swallowing, and tongue protrusion^(2,3)^. Speech-language-hearing therapy plays a significant role in rehabilitating these deleterious habits^(4,5)^.

Orthodontic expansion or rapid maxillary expansion (RME) is a dental treatment used to improve cases of transverse maxillary atresia. The main objective of the procedure in such cases is to increase the transverse dimension of the maxilla with palatal expanders such as the Hyrax, the McNamara Expander, and the Haas, a device supported on the teeth and mucosa^(6)^. RME is mainly recommended for children and adolescents under 15 years old, as skeletal resistance occurs after this age, requiring a combined surgical and orthodontic procedure in some cases^(2)^.

RME changes the maxilla, enabling adequate middle ear ventilation and balanced pressure on both sides of the tympanic membrane, enhancing the mobility and functioning of the ossicular chain^(7-9)^. Additionally, studies show a possible reduction in air-conduction hearing thresholds after maxillary expander activation, evidenced by a decrease in the air-bone gap – i.e., a difference of 15 or more dB HL between air and bone-conduction thresholds. Furthermore, these studies report improved middle ear function, verified by acoustic immittance measures^(9-18)^.

Thus, this study aimed to analyze whether RME affected middle ear function and the air-bone gap in children and adolescents. These effects were assessed at four different times: before RME, at the end of RME, and 3 and 6 months after RME.

## METHODS

This is a prospective, single-arm clinical trial. The outcome was the analysis of the effects on air-bone gaps and middle ear function, evaluated through tympanometry findings and acoustic reflex testing. Data were collected on four occasions: 1) before initiating RME (T0); 2) at the end of RME (T1); 3) 3 months after RME (T2); and 4) 6 months after RME (T3).

The research was submitted to and approved by the Research Ethics Committee of the Federal University of Rio Grande do Sul (UFRGS). Children and adolescents signed an informed assent form, and parents or guardians signed an informed consent form.

The target population comprised children and adolescents who attended the Orthodontics Clinic at UFRGS’s Dental School for RME. All children and adolescents who completed RME during the collection period (from March to November) were invited to participate. The inclusion criteria were children and adolescents submitted to RME. The exclusion criteria were children or adolescents who could not, for any reason, complete the evaluations stipulated in the protocol, with self-reported neurological and cognitive disorders, who underwent semi-RME, with cerumen occlusion, and/or with sensorineural hearing loss. No participant received speech-language-hearing therapy during data collection.

Participants and their guardians initially answered a medical history survey to gather sample characterization data, including hearing history, school history, and hearing and general health complaints. The procedures of each evaluation stage were explained in advance to participants and guardians.

They were evaluated at the following times: T0: before starting RME, T1: after completing RME, T2: 3 months after RME, and T3: 6 months after RME. Patients in retention or who had already finished treatment were contacted by phone to request a return for audiological examinations.

First, the external auditory canal was inspected to assess its condition. Children and adolescents without signs of obstruction or cerumen proceeded to auditory evaluations with air-conduction pure-tone audiometry (PTA) at 250, 500, 1000, 2000, 3000, 4000, 6000, and 8000 Hz and bone-conduction audiometry at 500, 1000, 2000, 3000, and 4000 Hz in a soundproof booth. The study used the World Health Organization classification to analyze air and bone-conduction thresholds^(19)^. Next, they underwent speech audiometry, including speech recognition threshold (SRT) and speech recognition percentage index (SRPI), analyzing responses according to Santos and Russo’s proposal^(20)^. PTA and speech audiometry were performed with a previously calibrated Interacoustics AD629 audiometer. Speech audiometry confirmed the PTA results in this study.

After PTA, the study measured acoustic immittance, including tympanometry and acoustic reflex testing, using the Interacoustics AT235. Tympanometry assesses tympanic membrane mobility and middle ear function by measuring the membrane’s ability to reflect sound in response to gradual pressure changes in the ear canal. Static and dynamic compliance were assessed, and curves were characterized according to the classification by Jerger et al.^(21)^. Normal values included static compliance between 0.3 and 1.6 ml and peak pressure between +50 and -100 daPa. Acoustic reflex is an involuntary contraction of the middle ear muscles in response to intense sound stimuli, generally 70-100 dB above the person’s air-conduction threshold. This reflex represents the lowest sound intensity capable of triggering the middle ear's protective mechanism, requiring the peripheral and central auditory system's structural and functional integrity at the brainstem level. The presence or absence of ipsilateral and contralateral acoustic reflexes was assessed at 500, 1000, 2000, and 4000 Hz^(21)^.

Data were analyzed using the Statistical Package for the Social Sciences 26 (SPSS 26). The study described quantitative variables by mean and standard deviation and categorical variables by absolute and relative frequencies. The Student’s t-test compared groups with normal distribution. Results were considered significant when p < 0.05, with a 95% confidence interval.

## RESULTS

The sample had 18 children and adolescents, 10 males and 8 females, aged 7 to 14 years, with a mean age of 10±1.7 years. Two children from the initial sample were excluded for not attending the T3 evaluation. The sample had a normal distribution (p > 0.05). There was no statistically significant difference (p > 0.05) between ears concerning the frequencies tested in PTA, reduction of the air-bone gap, reflex results, and tympanometry curves. Likewise, there was no significant difference (p > 0.05) between the participants’ sex and the study variables.

The tympanometry analysis (Table 1) showed that approximately 61% of the sample had changes in the tympanic-ossicular system before starting the RME, of which a percentage returned to normal by the end of the 6-month auditory follow-up.

**Table 1:** Tympanometric findings in children and adolescents undergoing rapid maxillary expansion (RME) (n = 18)

**Tympanometry (n = 18)**	**T0 n (%)**	**T1 n (%)**	**T2 n (%)**	**T3 n (%)**	**p-value** *
Type A	7 (38.89)	13 (72.22)	17 (94.44)	18 (100)	0.013
Type C	6 (33.33)	4 (22.22)	1 (5.56)	-	0.019
Type As	3 (16.67)	1 (5.56)	-	-	0.028
Type B	2 (11.11)	-	-	-	0.016

* *Student’s t-test*

**Caption:** RME = rapid maxillary expansion; T0 = before initiating RME; T1 = at the end of RME; T2 = 3 months after RME; T3 = 6 months after RME. Tympanometry types A, B, C, and As, according to the classification by Jerger et al.^(21)^

The investigation of ipsilateral and contralateral acoustic reflexes found they were absent in 66.67% of patients in both ears before RME, whereas all patients had ipsilateral and contralateral acoustic reflexes in both ears 6 months after RME (T3) (Table 2). The study also found a mean 18.8 dB difference in the air-bone gap from T0 to T3. Table 3 shows a reduction in air-bone gaps starting from T1 and an absence of gaps at T2 and T3 in all children and adolescents who underwent RME.

**Table 2:** Analysis of the presence or absence of ipsilateral and contralateral acoustic reflexes in children and adolescents undergoing rapid maxillary expansion (n = 18)

**Acoustic Reflexes (n = 18)**	**T0 n (%)**	**T1 n (%)**	**T2 n (%)**	**T3n (%)**	**p-value** *
Ipsilateral AR present in both ears	6 (33.33)	12 (66.67)	17 (94.44)	18 (100)	p < 0.001
Contralateral AR present in both ears	6 (33.33)	12 (66.67)	17 (94.44)	18 (100)	p < 0.001
Ipsilateral AR absent in both ears	12 (66.67)	6 (33.33)	1 (5.56)	0 (0)	p < 0.001
Contralateral AR absent in both ears	12 (66.67)	6 (33.33)	1 (5.56)	0 (0)	p < 0.001

* *Student’s t-test*

**Caption:** AR = acoustic reflexes; (%) = percentage; (n) = number of children and adolescents in the sample; T0 = before initiating RME; T1 = at the end of RME; T2 = 3 months after RME; T3 = 6 months after RME

**Table 3:** Analysis of air-bone gaps in children and adolescents undergoing rapid maxillary expansion (RME)

**Air-bone gaps(n = 18)**	**T0 n (%)**	**T1 n (%)**	**T2 n (%)**	**T3 n (%)**	**p-value** *
Presence of gap	11 (61.11)	5 (27.78)	0 (0.0)	0 (0.0)	p < 0.001
Absence of gap	7 (38.89)	13 (72.22)	18 (100)	18 (100)	p < 0.001

* *Student’s t-test*

**Caption:** RME = rapid maxillary expansion; air-bone gap = a difference greater than 15 dB HL between air and bone-conduction thresholds; (%) = percentage; (n) = number of children in the sample; T0 = before initiating RME; T1 = at the end of RME; T2 = 3 months after RME; T3 = 6 months after RME

Regarding acoustic immittance measures, this study showed that 66.67% of participants lacked acoustic reflexes in both ears before RME. Also, the tympanometry of 61.11% of the children and adolescents suggested middle ear impairment – type C was the most common among them (33.33%). Nevertheless, all individuals in the sample had type A tympanometry 3 months after removing the expander (T2) and acoustic reflexes in both ears 6 months after RME (T3). These acoustic immittance findings demonstrate that RME has significant effects on middle ear function, helping restore its integrity and especially the Eustachian tube function.

## DISCUSSION

RME is indicated for correcting transverse maxillary deficiencies, primarily in children and adolescents^(22)^. Systematic reviews on the subject suggest that RME can have positive effects on oral and nasopharyngeal anatomy, also improving hearing levels and middle ear function^(6,9,18)^. This study investigated the effects of maxillary expansion on middle ear function (through acoustic immittance analysis) and the air-bone gap in children and adolescents with maxillary transverse deficiency.

Most participants (61.11%) had type C tympanometry before RME, which may indicate improper Eustachian tube function^(23)^. Moreover, compared to similar studies^(8,24)^ that examined tympanometry, it was found that RME has significant effects on middle ear function. These studies observed that, after the procedure, acoustic immittance measures indicated the presence of acoustic reflexes and type A tympanometry in all sample subjects. One of these studies^(8)^ reported an increase in middle ear volume following maxillary expansion and the retention period. Additionally, the literature^(14,25)^ suggests that conductive hearing loss and transverse maxillary deficiency may be related when there is Eustachian tube dysfunction. The study further describes a positive effect of RME on hearing, stating that inadequate Eustachian tube function was more frequent in children and adolescents with maxillary deficiency.

Moreover, a recent study compared the effects of RME on children without orthodontic issues and with acute otitis media, Eustachian tube dysfunction, and maxillary constriction. The group with middle ear changes improved significantly in Eustachian tube function following RME^(26)^. The results of both studies were similar, as children and adolescents with Eustachian tube changes, indicated by type C tympanometry, improved their middle ear function after maxillary expansion. Thus, RME may be an effective treatment to prevent middle ear changes such as otitis media and Eustachian tube dysfunction, common in children with anatomical maxillary issues. Besides being a procedure with quick results, RME impacts the levator and tensor veli palatini muscles, helping to restore Eustachian tube function.

Regarding PTA findings, the abnormal air-conduction thresholds improved in both ears after using the expander, with a significant reduction in the air-bone gap (p < 0.001). These results corroborate findings in the scientific literature^(8,11,12,15-18)^ that indicate significantly improved air-conduction thresholds in both ears during and after expander activation, reducing the air-bone gap.

Another study involving non-syndromic groups and patients with Down syndrome demonstrated that maxillary expansion can reduce the incidence of infections such as otitis media, tubal dysfunction, and tonsillitis, contributing to efficient nasal breathing^(15)^.

There was no statistically significant difference between the left and right ears. However, there was a significant association between sex, age, and PTA and acoustic immittance findings. Furthermore, the number of participants may have been a limiting factor in the study.

The research results align with findings in the literature, demonstrating that children and/or adolescents affected by anatomical maxillary changes such as transverse maxillary atresia benefit from RME. This procedure acts on the median palatine structure, nasal floor, and elongation of the levator and tensor veli palatini muscles, thereby helping restore proper Eustachian tube function and potentially preventing middle ear changes^(2,27)^.

This study found that RME decreases the air-bone gap due to improved Eustachian tube function and nasopharyngeal tissues. However, further studies should include larger samples of this population to investigate and expand the scientific evidence regarding the effects of RME on the hearing of children and adolescents. Additionally, the lack of a control group – as all subjects seeking orthodontic treatment for maxillary atresia undergo palatal expansion – may have influenced the results of this research. Nevertheless, despite these limitations, and although RME is not indicated as a treatment for hearing loss, these findings indicate that such an approach should be considered for individuals with both maxillary expansion and conductive hearing loss, as RME can help restore middle ear function and improve air-conduction thresholds in children and adolescents.

## CONCLUSION

The study found that RME gradually reduced air-bone gaps and improved middle ear function in children and adolescents in the sample with transverse maxillary atresia.
