# Supplementary material for: Effects of rapid maxillary expansion on auditory thresholds and middle ear functioning
Source: Codas. 2025 Jan 27;37(1):e20230343. doi: 10.1590/2317-1782/e20230343en (PMC11781362; doi:10.1590/2317-1782/e20230343en)
Supplement: Translation

# Translation: Efeitos da expansão rápida de maxila nos limiares auditivos e funcionamento da orelha média

**DOI:** 10.1590/2317-1782/e20230343pt

**Authors:** Cassiele Fontoura Moraes, Erissandra Gomes, Rebeca Cardona Santa Helena, Pricila Sleifer

**Affiliations:** 1 Universidade Federal do Rio Grande do Sul – UFRGS - Porto Alegre (RS), Brasil.; 2 Faculdade de Odontologia, Universidade Federal do Rio Grande do Sul – UFRGS - Porto Alegre (RS), Brasil.; 3 Universidade Federal do Rio Grande do Sul – UFRGS - Porto Alegre (RS), Brasil.; 4 Instituto de Psicologia, Serviço Social, Saúde e Comunicação Humana, Universidade Federal do Rio Grande do Sul – UFRGS - Porto Alegre (RS), Brasil.

**Keywords:** Audição, Perda Auditiva, Técnica de Expansão Palatina, Maxila, Crianças, Adolescentes

## Abstract

**Objetivo:**

Verificar se a expansão rápida de maxila (ERM) causa efeitos no funcionamento da orelha média e nos gaps aéreo-ósseos em crianças e adolescentes.

**Método:**

Ensaio clínico de braço único, com coleta de dados em quatro momentos: antes de iniciar a ERM (T0), ao finalizar a ERM (T1), três meses após finalizar a ERM (T2) e seis meses após realizar a ERM (T3). A avaliação audiológica, realizada nos quatro momentos, foi composta pela meatoscopia, audiometria tonal liminar e vocal, timpanometria e pesquisa dos reflexos acústicos.

**Resultados:**

18 crianças e adolescentes atenderam os critérios de elegibilidade. Houve redução dos gaps aéreo-ósseos e melhora no funcionamento da orelha média, ao longo do acompanhamento, entre T0, T1, T2 e T3. Três meses após a finalização da ERM, em T2, todos os pacientes apresentaram curvas timpanométricas tipo A e seis meses após a ERM, em T3, houve ausência de gap aéreo-ósseo e reflexos acústicos ipsilaterais e contralaterais presentes em toda a amostra.

**Conclusão:**

Verificou-se na amostra estudada que a ERM promoveu gradativa redução dos *gaps* aéreo-ósseos e adequado funcionamento de orelha média em crianças e adolescentes com atresia transversal de maxila.

## INTRODUÇÃO

A atresia transversal da maxila é considerada uma anomalia dentofacial onde ocorre uma diminuição do diâmetro do arco maxilar, no sentido transversal^(1,2)^. Essa deficiência tem como principais fatores etiológicos a respiração oral, hábitos deletérios como a sucção digital e/ou de chupetas, deglutição adaptada/atípica e interposição lingual^(2,3)^. Destaca-se a importância do fonoaudiológico na reabilitação desses hábitos deletérios^(4,5)^.

Um dos tratamentos odontológicos utilizados para melhora nos quadros de atresia transversal da maxila é a Expansão Ortodôntica ou Expansão Rápida da Maxila (ERM). Nestes casos, o principal objetivo do procedimento consiste em melhorar a dimensão transversal da maxila, utilizando os expansores palatinos, como o Hyrax, o Disjuntor de McNamara e o Haas, um aparelho dentomucossuportado^(6)^. Indica-se a realização da ERM principalmente em crianças e adolescentes menores de 15 anos, porque após essa idade ocorre uma resistência esquelética e, em alguns casos, é necessária a associação de um procedimento cirúrgico combinado ao ortodôntico^(2)^.

A ERM provoca modificações na maxila, possibilitando a ventilação adequada da orelha média, fazendo com que seja possível equilibrar a pressão em ambos os lados da membrana timpânica e permitindo que a cadeia timpanossicular se movimente e funcione com maior efetividade^(7-9)^. Além disso, estudos demonstram que, após o período de ativação do aparelho expansor de maxila, pode haver redução nos limiares auditivos aéreos, constatado pela redução do *gap* aéreo-ósseo, que é considerado a diferença maior ou igual a 15 dBNA, entre os limiares aéreos e ósseos. Outrossim, referem um melhor funcionamento de orelha média, que pode ser verificado pelos achados das medidas de imitância acústica^(9-18)^.

À vista disso, este trabalho analisou se a ERM provocou efeitos no funcionamento da orelha média e no *gap* aéreo-ósseo, em crianças e adolescentes. Realizou-se a verificação de tais efeitos em quatro momentos distintos: antes da ERM, quando finalizada a ERM, após os três e seis meses, respectivamente.

## MÉTODO

Trata-se de um ensaio clínico de braço único, de abordagem prospectiva. O desfecho foi a análise dos efeitos provocados nos *gaps* aéreo-ósseos e no funcionamento da orelha média (analisados por meio dos achados da curva timpanométrica e da pesquisa dos reflexos acústicos). A coleta de dados foi realizada em quatro ocasiões: 1) antes de iniciar a ERM (T0); 2) ao finalizar a ERM (T1); 3) três meses após finalizar a ERM (T2) e 4) seis meses após realizar a ERM (T3).

A pesquisa foi submetida e aprovada pelo Comitê de Ética em Pesquisa da Universidade Federal do Rio Grande do Sul (UFRGS). As crianças e adolescentes assinaram o Termo de Assentimento Livre e Esclarecido, e os pais, mães e/ou responsáveis assinaram o Termo de Consentimento Livre e Esclarecido.

A população alvo constitui-se de crianças e adolescentes que frequentaram a Clínica de Ortodontia da Faculdade de Odontologia da UFRGS e que foram submetidos a ERM por opção de tratamento ortodôntico. Todas as crianças e adolescentes que realizaram a ERM no período da coleta foram eleitas e convidadas a participarem do estudo, durante os meses de março a novembro. Os critérios de inclusão foram crianças e adolescentes que realizaram o procedimento da ERM. Com relação aos critérios de exclusão, estes foram: crianças ou adolescentes que não compreenderam ou não conseguiram, por qualquer razão, realizar as avaliações estipuladas no protocolo, com doenças neurológicas e cognitivas autodeclaradas, que realizaram a expansão semi-rápida de maxila, que apresentaram tampão de cerume na orelha e/ou que apresentaram perda auditiva sensorioneural. Cabe ressaltar que nenhum participante realizou terapia fonoaudiológica durante a coleta de dados.

Inicialmente os participantes e seus responsáveis responderam a uma anamnese visando a obtenção de dados para a caracterização da amostra, informações relacionadas ao histórico auditivo, histórico escolar, queixas auditivas e de saúde geral. Para a realização dos procedimentos foram fornecidas, previamente, orientações para os responsáveis e para as crianças e adolescentes sobre cada etapa das avaliações.

As avaliações foram realizadas nos seguintes tempos: T0: antes do paciente iniciar a ERM, T1: quando o paciente finalizou a ERM, T2: três meses após a ERM e T3: seis meses após a ERM. Nos casos de pacientes que ficaram em período de contenção ou que já haviam finalizado o tratamento, o contato foi realizado pelo telefone, solicitando o retorno para realização dos exames audiológicos.

Primeiramente, foi realizada a inspeção do meato acústico externo, para verificar suas condições. As crianças e adolescentes que não apresentaram sinais de obstrução ou presença de cerúmen foram encaminhadas para as avaliações auditivas. A seguir, em cabine acústica, foi realizada a audiometria tonal liminar (ATL), por via aérea, nas frequências de 250, 500, 1000, 2000, 3000, 4000, 6000 e 8000Hz, e, também, por via óssea, nas frequências de 500, 1000, 2000, 3000 e 4000Hz. Para análise dos limiares aéreos e ósseos, foi utilizada a classificação da Organização Mundial da Saúde^(19)^. Na sequência, foi realizada a audiometria vocal, com pesquisa do limiar de reconhecimento de fala (LRF) e índice percentual de reconhecimento de fala (IPRF) e as respostas foram analisadas conforme proposto por Santos e Russo^(20)^. O audiômetro utilizado para realização da ATL e da audiometria vocal é da marca Interacoustics, modelo AD629, previamente calibrado. Na presente pesquisa, a audiometria vocal foi realizada com intuito de confirmar os resultados obtidos na ATL.

Após concluída a ATL, foram realizadas as medidas de imitância acústica (MIA), composta pela pesquisa das curvas timpanométricas e a pesquisa de reflexos acústicos, ambos realizados com equipamento AT235 da marca Interacoustics. A timpanometria é o método utilizado para avaliação da mobilidade da membrana timpânica e das condições funcionais de orelha média, onde é medida a capacidade que a membrana tem de refletir um som em resposta a graduais modificações de pressão no mesmo conduto. Foram pesquisadas as complacências estática e dinâmica, após a curva foi traçada e caracterizada de acordo com classificação proposta por Jerger et al.^(21)^. Cabe ressaltar que foram considerados como valores de normalidade: complacência estática entre 0,3 e 1,6 ml e pressão do pico entre +50 e -100daPa. O reflexo acústico é uma contração involuntária dos músculos da orelha média em resposta a um estímulo sonoro intenso, que geralmente acontece de 70 a 100 dB acima do limiar de via aérea do indivíduo. Ele representa a menor intensidade de um som capaz de desencadear o mecanismo de proteção da orelha média frente a sons intensos, sendo essencial a integridade estrutural e funcional do sistema auditivo periférico e central em nível de tronco encefálico. Foram pesquisados os reflexos acústicos ipsilaterais e contralaterais nas frequências de 500Hz, 1000Hz, 2000Hz e 4000Hz e analisados quanto à presença ou ausência^(21)^.

Os dados foram analisados no Software *Statistical Package for the Social Sciences* 26 (SPSS 26). As variáveis quantitativas foram descritas por média e desvio padrão; as variáveis categóricas por frequências absolutas e relativas. Foi utilizado o Teste t, de *Student*, para a comparação dos grupos com distribuição normal. Os resultados são considerados significativos quando p<0,05, com intervalo de confiança de 95%.

## RESULTADOS

A amostra foi composta por 18 crianças e adolescentes, 10 do gênero masculino e 8 do gênero feminino, com idades entre 7 e 14 anos, com média de 10±1,7 anos, da amostra inicial duas crianças foram excluídas por não comparecerem na avaliação do T3. A amostra apresentou distribuição normal (p>0,05). Não houve diferença estatisticamente significativa (p>0,05) na comparação entre as orelhas, em relação às frequências testadas na ATL, redução do *gap* aéreo-ósseo, resultados dos reflexos e curvas timpanométricas. Além disso, não houve diferença significativa (p>0,05) entre os gêneros dos participantes e as variáveis pesquisadas.

Nos resultados da pesquisa da curva timpanométrica (Tabela 1), cerca de 61% da amostra apresentou alteração no sistema tímpano-ossicular antes de iniciar a ERM, percentual que atingiu à normalidade ao final dos seis meses de acompanhamento auditivo.

**Tabela 1:** Achados das curvas timpanométricas em crianças e adolescentes submetidos a ERM (n=18)

**Curvas Timpanométricas (n=18)**	**T0 n (%)**	**T1 n (%)**	**T2 n (%)**	**T3 n (%)**	**p-valor** *
Curva Tipo A	7 (38,89)	13 (72,22)	17 (94,44)	18 (100)	0,013
Curva Tipo C	6 (33,33)	4 (22,22)	1 (5,56)	-	0,019
Curva Tipo Ar	3 (16,67)	1 (5,56)	-	-	0,028
Curva Tipo B	2 (11,11)	-	-	-	0,016

* *Test T Student*

**Legenda:** ERM = expansão rápida da maxila; T0 = antes de iniciar a ERM; T1 = ao finalizar a ERM; T2 = três meses após finalizar a ERM; T3 = seis meses após realizar a ERM. Curvas timpanométricas tipo A, B, C e Ar, conforme classificação de Jerger et al.^(21)^

Em relação à pesquisa dos reflexos acústicos ipsilaterais e contralaterais, antes da ERM, estes foram ausentes em 66,67% dos pacientes em ambas as orelhas, sendo que em seis meses após a ERM (T3) todos os pacientes apresentaram reflexos acústicos ipsilaterais e contralaterais presentes em ambas as orelhas (Tabela 2). Observou-se uma diferença de 18,8dB (média), no intervalo aéreo-ósseo, quando comparado T0 a T3. Na tabela 3, verificou-se redução dos *gaps* aéreo-ósseos a partir do T1 e ausência no T2 e T3, em todas crianças e adolescentes submetidos a ERM.

**Tabela 2:** Análise da presença ou ausência dos reflexos acústicos ipsilaterais e contralaterais em crianças e adolescentes submetidos a expansão rápida de maxila (n=18)

**Reflexos Acústicos (n=18)**	**T0 n (%)**	**T1 n (%)**	**T2 n (%)**	**T3n (%)**	**p-valor** *
Presença de RA ipsilateral em ambas as orelhas	6 (33,33)	12 (66,67)	17 (94,44)	18 (100)	p < 0,001
Presença de RA contralateral em ambas as orelhas	6 (33,33)	12 (66,67)	17 (94,44)	18 (100)	p < 0,001
Ausência de RA ipsilateral em ambas as orelhas	12 (66,67)	6 (33,33)	1 (5,56)	0 (0)	p < 0,001
Ausência de RA contralateral em ambas as orelhas	12 (66,67)	6 (33,33)	1 (5,56)	0 (0)	p < 0,001

* *Test T Student*

**Legenda:** RA = Reflexos Acústicos; (%) = porcentagem; (n) = número de crianças e adolescentes da amostra; T0 = antes de iniciar a ERM; T1 = ao finalizar a ERM; T2 = três meses após finalizar a ERM; T3 = seis meses após realizar a ERM

**Tabela 3:** Análise de *gaps* aéreo-ósseos em crianças e adolescentes submetidos a ERM

***Gaps* aéreo-ósseos (n=18)**	**T0 n (%)**	**T1 n (%)**	**T2 n (%)**	**T3 n (%)**	**p-valor** *
Presença de *gap*	11 (61,11)	5 (27,78)	0 (0,0)	0 (0,0)	p < 0,001
Ausência de *gap*	7 (38,89)	13 (72,22)	18 (100)	18 (100)	p < 0,001

* *Test T Student*

**Legenda:** ERM = expansão rápida de maxila; *gap* aéreo-ósseo = diferença maior que 15 dBNA entre os limiares aéreos e ósseos; (%) = porcentagem; (n) = número de crianças da amostra; T0 = antes de iniciar a ERM; T1 = ao finalizar a ERM; T2 = três meses após finalizar a ERM; T3 = seis meses após realizar a ERM

No que diz respeito às medidas de imitância acústica, este estudo mostrou que antes da ERM, 66,67% dos participantes apresentaram reflexos acústicos ausentes em ambas as orelhas. Com relação às curvas timpanométricas, verificou-se que 61,11% das crianças e adolescentes apresentaram curvas que sugerem comprometimento de orelha média, sendo a curva tipo C com maior ocorrência entre os indivíduos (33,33%). Passados 3 meses da retirada do expansor (T2), todos os indivíduos da amostra apresentaram curvas timpanométricas do tipo A. Seis meses após a ERM (T3), os mesmos apresentaram reflexos acústicos presentes em ambas as orelhas. Tais achados das MIA demonstram que a ERM provoca efeitos significativos no funcionamento da orelha média, ajudando a restabelecer sua integridade e, principalmente, a função da tuba auditiva.

## DISCUSSÃO

A ERM é um procedimento indicado para corrigir deficiências maxilares transversais, principalmente em crianças e adolescentes^(22)^. Revisões sistemáticas realizadas acerca do assunto apontam que a ERM pode trazer resultados positivos para a anatomia oral e da nasofaringe, o que provoca melhoras, também, nos níveis de audição e funcionamento da orelha média^(6,9,18)^. A presente pesquisa investigou os efeitos da disjunção maxilar, no funcionamento da orelha média (por meio da análise das medidas de imitância acústica) e no *gap* aéreo-ósseo, em crianças e adolescentes com atresia transversal de maxila.

No tocante às curvas timpanométricas, a maioria dos participantes (61,11%) apresentou curva C antes da ERM, o que pode indicar um inadequado funcionamento da tuba auditiva^(23)^. Ademais, em comparação com estudos semelhantes^(8,24)^ que realizaram a pesquisa da timpanometria, constatou-se que a ERM tem efeitos significativos no funcionamento da orelha média. Nesses estudos foi observado que, após o procedimento, os resultados das medidas de imitância acústica indicaram presença de reflexos acústicos e curvas timpanométricas do tipo A em todos os sujeitos da amostra. Em uma destas pesquisas^(8)^, foi relatado que o volume da orelha média aumentou depois da expansão maxilar e do período de retenção. Ademais, há relatos na literatura^(14,25)^ de que a perda auditiva condutiva e a atresia transversal de maxila podem estar relacionadas quando existe uma disfunção da tuba auditiva. O estudo ainda descreve um efeito positivo da ERM na audição, afirmando que o inadequado funcionamento da tuba auditiva foi mais frequente em crianças e adolescentes que tinham disjunção maxilar.

Ainda, em uma pesquisa recente, foram comparados os efeitos da expansão rápida da maxila em crianças sem alterações ortodônticas com crianças que apresentavam otite média aguda, disfunção da tuba auditiva e constrição maxilar. O grupo com alterações de orelha média revelou melhorias significativas nas funções da tuba auditiva após a ERM^(26)^. Os resultados de ambas as pesquisas foram semelhantes, visto que as crianças e adolescentes que manifestaram alterações na tuba auditiva, com presença de curvas timpanométricas do tipo C, apresentaram melhorias na função de orelha média após a disjunção maxilar. Dessa forma, percebeu-se que a expansão rápida de maxila pode ser considerada um tratamento aceitável para prevenir alterações de orelha média, tais como a otite média e disfunção tubária, que são recorrentes em crianças afetadas por alterações anatômicas da maxila. Além de ser considerado um procedimento com rápido resultado, a ERM atua nos músculos elevadores e tensor palatino, ajudando a restaurar a função da tuba auditiva.

Em relação aos achados da audiometria tonal liminar, observou-se uma melhora nos limiares auditivos aéreos alterados, com análise da redução de *gap* aéreo-ósseo (p<0,001) após uso do expansor em ambas as orelhas. Os resultados corroboram os achados da literatura científica^(8,11,12,15-18)^, os quais apontam que, tanto durante, quanto após o período de ativação do aparelho expansor, houve melhora significativa dos limiares auditivos aéreos em ambas as orelhas e, consequentemente, uma diminuição do *gap* aéreo-ósseo.

Além disso, um estudo envolvendo grupos não sindrômicos e pacientes com Síndrome de Down demonstrou que a expansão maxilar pode diminuir a incidência dessas infecções, tais como otite média, disfunção tubária e amigdalite, contribuindo para uma respiração nasal eficiente^(15)^.

Não houve diferença estatisticamente significante entre a orelha esquerda e a orelha direita e uma associação significante entre as variáveis sexo, idade e os achados da ATL e MIA. Além disso, acredita-se que o número de participantes possa ter sido um limitador do estudo.

Os resultados apresentados nesta pesquisa vão ao encontro dos achados na literatura, mostrando que crianças e/ou adolescentes acometidas por alterações anatômicas da maxila, como a atresia transversal de maxila, se beneficiam da expansão rápida de maxila, visto que este atua na estrutura palatina mediana, no assoalho nasal e no alongamento dos músculos elevador e tensor palatino, também auxiliando no restabelecimento da função adequada da tuba auditiva, podendo, assim, prevenir alterações de orelha média^(2,27)^.

Observou-se neste estudo que a ERM provoca diminuição do *gap* aéreo-ósseo devido ao melhor funcionamento da tuba auditiva e dos tecidos da nasofaringe. Contudo, sugere-se a realização de outros estudos nessa população, com amostras maiores, a fim de investigar e ampliar as evidências científicas acerca dos efeitos da ERM na audição de crianças e adolescentes. Além disso, devido à impossibilidade de um grupo controle, visto que todos os sujeitos que chegam para tratamento ortodôntico para atresia maxilar realizam a disjunção palatina, pode ter interferido nos resultados da presente pesquisa. Todavia, apesar das limitações citadas, e de não ser indicada a realização da expansão maxilar como tratamento para a perda auditiva, tais achados mostram que nos casos em que o indivíduo possui, concomitantemente, a disjunção maxilar e a perda auditiva condutiva, tal conduta deve ser levada em consideração, visto que a ERM pode ajudar a restabelecer a função de orelha média e contribuir na melhora dos limiares auditivos aéreos de crianças e adolescentes.

## CONCLUSÃO

Verificou-se na amostra estudada que a ERM promoveu gradativa redução dos *gaps* aéreo-ósseos e adequado funcionamento de orelha média em crianças e adolescentes com atresia transversal de maxila.
